# Therapeutic Potential of INM‐901 in Mitigating Alzheimer’s Disease Pathology: Insights from a Long‐term 5xFAD Mouse Model Study

**DOI:** 10.1002/alz70859_103609

**Published:** 2025-12-25

**Authors:** Rishi K Somvanshi, Shadi Madani, Sneha Singh, Sarah Ng, Ashish Sarkar, Marjan Barazandeh, James Kealey, James Craig, Sapna Padania, Eric A Adams, Michael Woudenberg, Eric Hsu, Ujendra Kumar

**Affiliations:** ^1^ North Island College, Courtenay, BC Canada; ^2^ The University of British Columbia, Vancouver, BC Canada; ^3^ BayMedica LLC, South San Francisco, CA USA; ^4^ InMed Pharmaceuticals Inc, Vancouver, BC Canada

## Abstract

**Background:**

Alzheimer's Disease (AD) is a neurodegenerative condition characterized by cognitive and sensorimotor deficits, affecting over 6.9 million people in the US, with an annual economic impact of over $700 billion in direct and indirect healthcare costs. While current treatments such as donepezil, and memantine manage symptoms, they do not halt disease progression. Moreover, amyloid beta (Aβ) antibody therapy faces challenges, including limited efficacy in advanced disease stages, infusion‐related reactions, and high treatment costs. Cannabinoids have shown potential in alleviating Aβ toxicity, reducing tau phosphorylation, and suppressing inflammation via CB1 and CB2 receptors, supporting neuronal viability. Therefore, in this study, we investigated the effects of a novel synthetic cannabinoid analogue INM‐901 on Aβ‐induced toxicity and disease progression using the 5xFAD mice.

**Method:**

Male 5xFAD mice, which exhibit AD‐like pathology, including accelerated Aβ‐plaque accumulation, inflammation, neurodegeneration, and deficits in cognitive and motor functions, were treated (intra‐peritoneally) with INM‐901 at 15 or 30 mg/kg twice‐weekly for 7 months. Control groups, including non‐transgenic and 5xFAD mice, received vehicle‐treatment. Behavioral tests, including the Open‐field (OFT), Zero Maze, Barnes Maze, and Acoustic Startle Response, were conducted post‐treatment. Brain tissue and plasma samples were collected and analyzed via RNAseq, immunohistochemistry, western blotting, and multiplex assay to assess the effects of INM‐901 on AD‐related genes and protein expression.

**Result:**

INM‐901 treatment reversed changes in anxiety‐like behavior in the Zero Maze and OFT, as well as improved spatial learning and memory in the Barnes Maze. INM‐901 treated mice also exhibited improved acoustic startle response (%PPI), indicating enhanced auditory function. RNAseq showed decreased expression of several inflammatory genes that were upregulated in the 5xFAD mice, while multiplex assays revealed reduced levels of pro‐inflammatory cytokines and neurodegeneration marker neurofilament light chain (NfL). Immunohistochemistry demonstrated a reduction in Aβ‐aggregation, as well as changes in CB2R expression, highlighting the neuroprotective and anti‐inflammatory effects of INM‐901.

**Conclusion:**

INM‐901 treatment reversed several behavioral changes, improved auditory deficits, decreased Aβ‐aggregation, and modulated inflammatory and neuritogenesis markers in 5xFAD mice. These findings highlight the potential of INM‐901 as a therapeutic candidate for AD and provide a basis for further evaluation in tauopathy and inflammatory neurodegenerative models.

